# An Updated Meta-analysis: Similar Clinical Efficacy of Anterior and Posterior Approaches in Peroral Endoscopic Myotomy (POEM) for Achalasia

**DOI:** 10.1155/2022/8357588

**Published:** 2022-04-11

**Authors:** Weina Jing, Xinyue Luo, Jinlin Yang, Junchao Wu, Yuxiang Chen, Kai Deng

**Affiliations:** ^1^Department of Gastroenterology & Hepatology, West China Hospital, Sichuan University, Chengdu, 610041 Sichuan, China; ^2^Sichuan University-Oxford University Huaxi Gastrointestinal Cancer Centre, West China Hospital, Sichuan University, Chengdu, 610041 Sichuan, China

## Abstract

**Introduction:**

Currently, there are few studies on the efficacy of peroral endoscopic myotomy (POEM) in the anterior or posterior approach; however, limited studies have shown contradictory findings. Thus, the goal was to obtain more quantitative and objective outcomes and further compare the clinical efficacy of these two approaches in this meta-analysis.

**Methods:**

A comprehensive search of PubMed, Embase, Cochrane Library, and Web of Science was conducted to find studies relevant to POEM. The retrieval time was from database inception to September 2021. Studies reporting the effects of POEM according to the anterior or posterior approach were included. STATA 16.0 was used to perform statistical analysis, mainly comparing the quantitative objective indicators (lower esophageal sphincter (LES) pressure and Eckardt scores, etc.) in anterior and posterior approaches by meta-analysis.

**Result:**

A total of 19 studies with 1261 patients were finally included. Except for shorter procedure time in the posterior approach, other factors (pooled difference of LES pressure, Eckardt scores, clinical success, length of total myotomy, hospital stays, gastroesophageal reflux (GERD), and adverse event) were compared, and all above confirmed that there is no difference between anterior and posterior approaches, and the safety of POEM is ensured. In addition, both anterior and posterior myotomy can improve LES pressure and Eckardt scores, and the difference in anterior and posterior myotomy was unconspicuous.

**Conclusion:**

The terms of the pooled difference in LES pressure, Eckardt scores, and other factors (clinical success, length of total myotomy, hospital stays, GERD, adverse events, and procedure time) seemed to be similar for the anterior and posterior approaches. However, the further prognosis after POEM via anterior and posterior approaches needs to be answered in the future.

## 1. Introduction

Achalasia (AC), which means “nonrelaxing” in Greek, is a primary disorder of esophageal motility with the main features of lower esophageal sphincter (LES) relaxation disorder and reduced esophageal peristalsis [1]. Its typical clinical manifestations, including severe dysphagia, regurgitation, retrosternal pain, and weight loss, affect the quality of life of patients. Previous epidemiology suggested that it was a rare disease affecting only 1 in 100,000 people [2, 3]; nevertheless, the incidence rate in recent years has increased to 2 to 3 times [4].

The current treatments for achalasia include pneumatic dilation (PD), botulinum toxin injection (BTI), laparoscopic Heller myotomy (LHM), and peroral endoscopic myotomy (POEM). Because it is an incurable disease, the aim of the treatment is to remit LES relaxation disorder and lower LES pressure to relieve the symptoms of obstruction [5]. As recurrent dysphagia of PD and BTI often requires repeated treatment, LHM and POEM have become the main treatment methods because of their better efficacy [6–9].

POEM has been used more widely over the past decade because of its confirmed safety and efficiency [10, 11]. However, recent data showed that the incidence of postprocedure gastroesophageal reflux (GERD) of POEM can be up to 40%, which was higher than that of LHM [12]. Some studies have indicated that the myotomy length, achalasia subtype, and history of previous treatment had no effect on the occurrence of postprocedure GERD in POEM. A circular myotomy of the anterior approach might lessen the postprocedure GERD of POEM [13]. Through theoretical analysis, the anterior approach, in the 2-3 o'clock position, is easier than the posterior approach and has a lesser risk of damage to sling muscle fibers and the angle of His, which might be more beneficial to the antireflux mechanism of the esophagus [5, 14]. Nevertheless, a prior study showed that the rate of postprocedure GERD of POEM, clinical success, and adverse events were almost the same in both anterior and posterior approaches [14], which was contradicted by other studies.

This prior study [14], as a currently available estimate in the literature with respect to the clinical outcomes of anterior and posterior myotomy in POEM, compared clinical success, GERD, and adverse events between anterior and posterior myotomy. Nevertheless, these outcomes had more subjectivity since they were not based on quantitative objective indicators, such as LES pressure and Eckardt score.

Therefore, based on a previous study [14], the purpose of this study was to analyze objective indicators to obtain more quantitative and objective outcomes and update the analysis data through a meta-analysis of studies grouping POEM according to anterior and posterior approaches and to further compare the clinical efficacy of these approaches.

## 2. Methods

### 2.1. Search Strategy

For this meta-analysis, a comprehensive search of several databases, including PubMed, Embase, Cochrane Library, and Web of Science, from database inception to September 2021 was conducted. The search string consisted of the following keywords: “Achalasia”, “Achalasia, Esophageal”, “POEM”, and “peroral endoscopic myotomy”, as detailed in Appendix 1. In addition, references to the evaluated articles were checked to identify additional studies.

### 2.2. Selection Criteria

In this meta-analysis, the two authors (WNJ and XYL) screened the articles that needed to be evaluated together, and the screening process was carried out strictly according to the following procedures and standards. All conflicts between the two researchers were resolved by conference. First, irrelevant literature was eliminated by title and abstract. Then, according to the inclusion and exclusion criteria, studies were included through full-text reading. The inclusion criteria were as follows: (1) adults (participants aged ≥18 years) diagnosed with achalasia by clinical symptoms, barium contrast, or esophageal manometry; (2) POEM in the anterior or posterior approach; (3) outcomes included Eckardt score, LES pressure, clinical success rate, incidence of complications, and incidence of GERD; and (4) original study. The exclusion criteria were as follows: (1) the effect of POEM was not analyzed according to the approach; (2) the study population was less than 20 patients; (3) animal studies; (4) the study data were not available; and (5) studies not published in English. If there were multiple studies from the same cohort for the same experiment, data from the most recent and/or most appropriate comprehensive single report were included.

### 2.3. Data Extraction and Quality Evaluation

According to a standardized data extraction form that had been previously formulated, the following information was independently extracted by two authors (WNJ and XYL): first author, year of publication, country, journal, study design, study period, site of myotomy, range of ages, number of patients, gender ratio, follow-up duration (months), type of achalasia, course of disease (months), prior treatment/intervention, pre- and postoperative LES pressures (mmHg), pre- and post-POEM Eckardt scores, procedure time (minutes), length of myotomy (cm), hospital stays (day), number of clinical successes after POEM at 12 months and >12 months, postprocedure GERD evidenced by esophagogastroduodenoscopy (EGD), and adverse events.

The Newcastle–Ottawa Scale (NOS) for cohort studies was used to assess the quality of cohort studies [15], while the Jadad score was used to assess the quality of randomized controlled trials (RCTs) [16]. The NOS quality score contained 8 questions, and the Jadad score consisted of 4 questions, as detailed in Supplementary Table 1-2. Two authors (WNJ and XYL) independently evaluated the eligibility of the included studies. In case of disagreement, a third author (KD) would participate in the discussion.

### 2.4. Data Analysis

In this meta-analysis, a random-affects model was used to calculate the pooled estimates in each case according to the methods suggested by Der Simonian and Laird [17]. Before statistical analysis, if the incidence of an outcome was zero in a study, a continuity correction of 0.5 was added to the number of incident cases [18].

### 2.5. Outcomes Assessed

#### 2.5.1. Primary Outcome

Quantitative indicator consists of pooled difference in LES pressure and Eckardt scores before and after POEM in the anterior approach and posterior approach.

#### 2.5.2. Secondary Outcomes

1. Length of total myotomy in the anterior approach and posterior approach
2. Hospital stays in the anterior approach and posterior approach
3. Overall clinical success after POEM at 12 months and >12 months in the anterior approach and posterior approach
4. Pooled occurrence of adverse events after POEM in the anterior approach and posterior approach
5. Pooled occurrence of GERD events after POEM in the anterior approach and posterior approach (according to EGD findings)
6. Procedure time in the anterior approach and posterior approach

The assessment methodology and definitions are as follows:

1. The pooled difference in LES pressure is calculated by subtracting the pre-POEM LES pressure from the post-POEM LES pressure
2. Pooled difference of Eckardt scores is calculated by post-POEM Eckardt scores minus pre-POEM Eckardt scores
3. In the included studies, clinical success was defined as achieving an Eckardt score ≤ 3 postprocedure [19],
4. Adverse events were defined as mild, moderate, or severe events, as reported by the American Society for Gastrointestinal Endoscopy (ASGE) lexicon [20]
5. Postprocedure GERD was evaluated by EGD findings based on the Los Angeles classification of esophagitis (> A) [21]

Metaregression analyses were used to evaluate whether the length of total myotomy, proportion of type II achalasia, prior treatments (PBD, EBTI, and Heller's myotomy), course of disease, and length of follow-up time had any effect on the primary outcomes.

### 2.6. Validation of Meta-analysis Results

#### 2.6.1. Heterogeneity

The *I*^2^ measure from the netmeta statistical package was used to investigate the heterogeneity. *I*^2^ values <30% are low, values of 30-60% are moderate, values of 61%-75% are substantial, and values >75% indicate considerable heterogeneity [22].

#### 2.6.2. Publication Bias

The funnel plot and the Egger test were used to identify publication bias qualitatively and quantitatively [23]. If there was any publication bias, the trim and fill method of Duval and Tweedie was used to perform the adjustment [24]. Publication bias for the RCTs was not ascertained separately, since the number of studies was <10.

*P* values < 0.05 on both tails were considered statistically significant in all tests. The Preferred Reporting Items for Systematic Reviews and Meta-Analyses (PRISMA) guidelines [25] were followed to perform analysis and reporting, and the PRISMA checklist is shown in Appendix 2. All statistical procedures were performed using Stata (version 16.0).

## 3. Results

### 3.1. Study Selection and Quality of Included Studies

**Figure 1:**
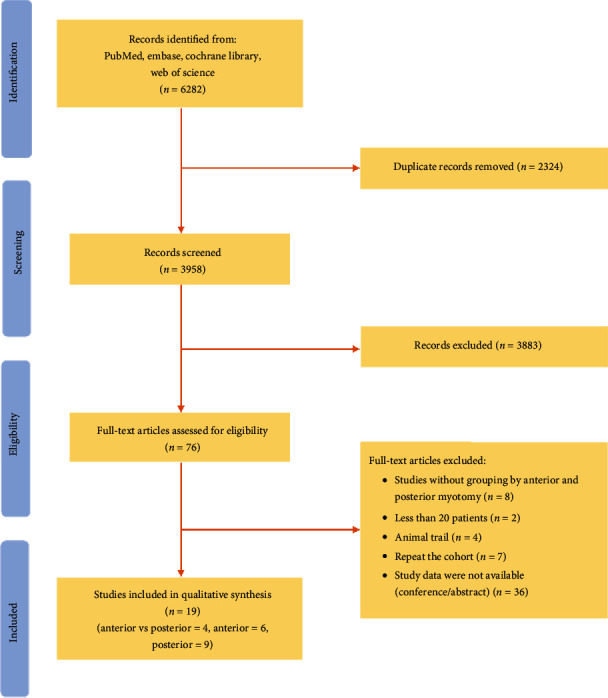
Study selection flow chart.

A total of 3958 studies were identified in this literature search after removing 2324 duplications. After screening the titles and abstracts, 3883 irrelevant studies were excluded. The remaining 76 full-length articles were identified, and 57 studies were excluded. Finally, 19 studies were included in this meta-analysis. These studies were published between 2016 and 2020. Six studies only reported the outcomes of POEM in the anterior approach [26–31], and nine studies only reported the outcomes of POEM in the posterior approach [32–40], while four studies compared outcomes of POEM via the anterior approach and posterior approach [41–44]. This meta-analysis included ten studies reporting outcomes with POEM via anterior myotomy and thirteen studies reporting outcomes with POEM via posterior myotomy. The flow chart of this literature search and final inclusions is illustrated in Figure 1. Seven studies were replicated in the cohort, and the most comprehensive recent studies were included [45–50].

This meta-analysis included three RCTs [41–43], of which two were considered low quality and one was considered high quality. Of the remaining 16 studies, 12 studies were considered high quality, while 4 studies were considered medium quality. Overall, 13 of 19 studies (68.4%) were considered high quality. The details of the NOS quality scores and Jadad scores are shown in Supplementary Table 1-2.

### 3.2. Population Characteristics

This meta-analysis finally included a total of 1261 patients in this analysis (606 patients in the anterior approach and 655 patients in the posterior approach). The age range was 33-63 years in the anterior approach and 38-68 years in the posterior approach. The male proportion was 55% in the anterior approach and 51% in the posterior approach. The follow-up duration of patients after POEM ranged from a minimum of 6 months to a maximum of 46.2 months. The baseline characteristics of the anterior approach and posterior approach were comparable, and the detailed characteristics of the included studies are summarized in Table 1.

## 4. Outcomes

### 4.1. Pooled Difference in LES Pressure

**Figure 2:**
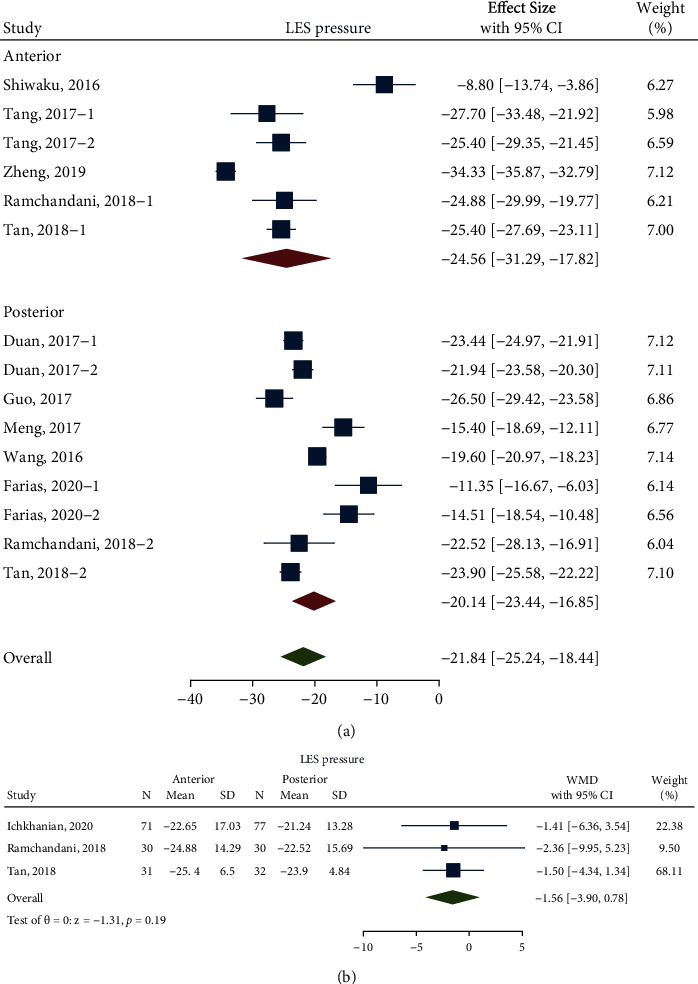
(a) Forest plot, difference in pre- and postoperative LES pressure before balancing baseline; (b) Forest plot, pre- and postoperative LES pressure difference between anterior and posterior approaches after balancing baseline Labels 1 and 2 were sectionalizations inside the study. They grouped these factors as follows: preoperative intervention/non-preoperative intervention (Tang, 2017), FTM/CM (Duan, 2017), Chagas/idiopathic (Farias, 2020), and anterior/posterior (Ramchandani, 2018; Tan, 2018).

The meta-analysis for the pooled difference in LES pressure comprised 10 studies with 574 patients (218 patients in the anterior approach and 356 patients in the posterior approach). The pooled difference in LES pressure via the anterior approach was -24.56 mmHg (95% confidence interval (CI) -31.29 to -17.82 mmHg; *n* = 5; *I*^2^ 96.25%) and that via the posterior approach was -20.14 mmHg (95% CI -23.44 to -16.85 mmHg; *n* = 7; *I*^2^ 94.72%), which showed no significant difference between anterior myotomy and posterior myotomy (*P* = 0.25) (Figure 2(a)). However, significant heterogeneity was observed (*I*^2^ = 96.58%; *n* = 10). Thus, metaregression analysis and sensitivity analysis were performed to determine the sources of heterogeneity. In the metaregression analysis based on length of total myotomy, the proportion of type II achalasia, prior treatments (PBD, EBTI, and Heller's myotomy), course of disease, and length of follow-up time did not show any effect on the previous outcome, as none of the two-tailed *P* values was less than 0.05 (Table 2). In the subsequent sensitivity analysis, none of the included studies was relevant to the heterogeneity, which shows the robustness of our results (Supplementary Fig. 6).

To eliminate the interference of baseline differences between studies on the results, better baseline level control was needed. Thus, further analysis was conducted in studies with a direct comparison between anterior and posterior approaches. Among all the included studies, 3 studies met the conditions of direct comparison because the patients were divided into anterior and posterior groups for comparison in these studies (including two RCTs [41, 43] and one cohort [44]). The analysis after balancing baseline showed that the pooled weighted mean difference (WMD) was -1.56 mmHg (95% CI -3.09 to 0.78 mmHg; *I*^2^ 0.00%; *n* = 3; *P* = 0.19) (Figure 2(b)), which still showed no significant difference between these two groups, and the significant heterogeneity disappeared.

### 4.2. Pooled Difference in Eckardt Scores

**Figure 3:**
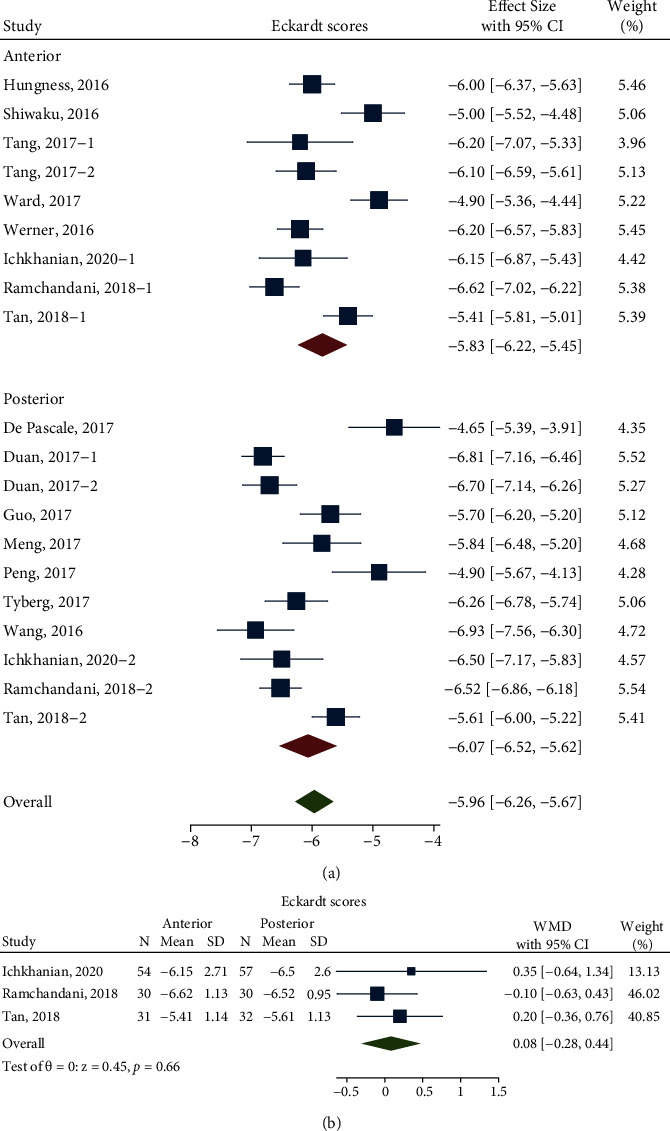
(a) Forest plot, difference in pre- and postoperative Eckardt scores before balancing baseline; (b) Forest plot, Differences in the pre- and postoperative Eckardt scores of the anterior and posterior approaches after balancing baseline Labels 1 and 2 were sectionalizations inside the study. They grouped these factors as follows: preoperative intervention/non-preoperative intervention (Tang, 2017), FTM/CM (Duan, 2017), Chagas/idiopathic (Farias, 2020), and anterior/posterior (Ramchandani, 2018; Tan, 2018).

The meta-analysis for the pooled difference of Eckardt scores comprised 15 studies with 937 patients (479 patients in the anterior approach and 458 patients in the posterior approach). The pooled difference in Eckardt scores via the anterior approach was -5.83 (95% CI -6.22 to -5.45; *n* = 8; *I*^2^ 83.15%) and that via the posterior approach was -6.07 (95% CI -6.52 to -5.62; *n* = 10; *I*^2^ 88.93%), which showed no significant difference between anterior myotomy and posterior myotomy (*P* = 0.44) with high heterogeneity (Figure 3(a)). To determine the sources of heterogeneity, metaregression analysis and sensitivity analysis were performed. Similarly, the metaregression analysis based on length of total myotomy, proportion of type II achalasia, prior treatments (PBD, EBTI, and Heller's myotomy), course of disease, and length of follow-up time showed no relevance between them and the heterogeneity, as shown in Table 2. The sensitivity analysis did not show any study relevant to the heterogeneity (Supplementary Fig. 6).

Likewise, a better balancing of the baseline characteristics was needed, and then, analysis of directed comparison was performed. The analysis of Eckardt scores after balancing baseline revealed that the pooled WMD was 0.08 (95% CI -0.28 to 0.44; *I*^2^ 0.00%; *n* = 3; *P* = 0.66), without heterogeneity (Figure 3(b)).

### 4.3. Length of Total Myotomy and Hospital Stays

**Figure 4:**
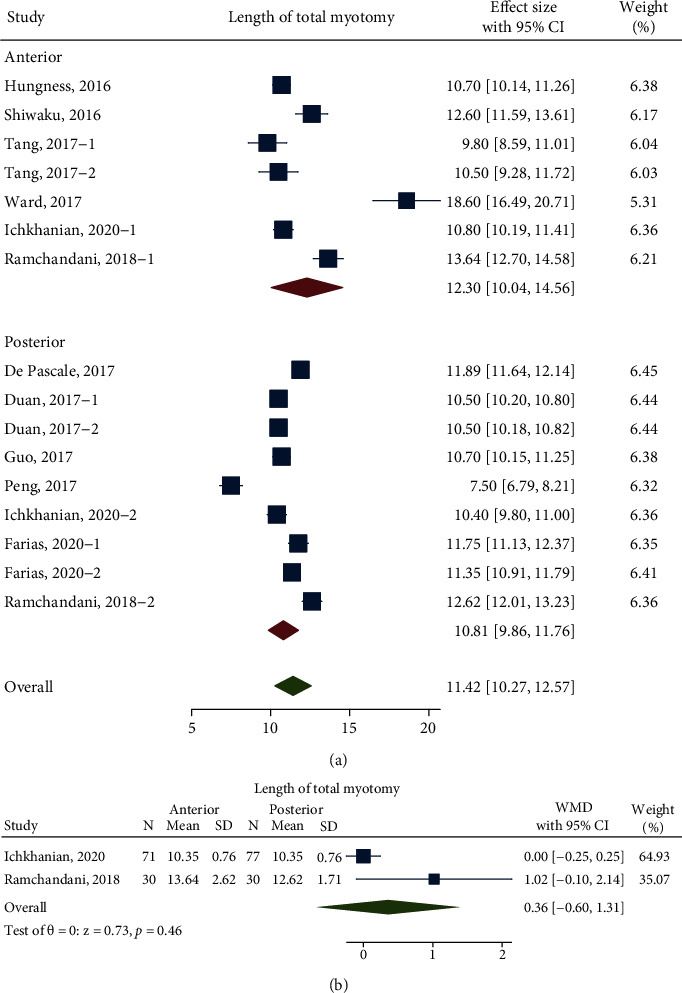
(a) Forest plot, Comparison of the length of total myotomy before balancing baseline; (b) Forest plot, Length of total myotomy difference between anterior and posterior approaches after balancing baseline Labels 1 and 2 were sectionalizations inside the study. They grouped these factors as follows: preoperative intervention/non-preoperative intervention (Tang, 2017), FTM/CM (Duan, 2017), Chagas/idiopathic (Farias, 2020), and anterior/posterior (Ramchandani, 2018; Tan, 2018).

With the meta-analysis of 11 studies including 741 patients (368 patients in the anterior approach and 373 patients in the posterior approach), the length of total myotomy was 12.30 cm (95% CI 10.04 to 14.56 cm; *n* = 6; *I*^2^ 97.70%) in the anterior approach and 10.81 cm (95% CI 9.86 to 11.76 cm; *n* = 7; *I*^2^ 97.80%) in the posterior approach. There was no significant difference between them (*P* = 0.23) (Figure 4(a)). After balancing the baseline characteristics of the studies by direct comparison, the pooled WMD was 0.36 cm (95% CI -0.60 to 1.31 cm; *I*^2^ 67.14%; *n* = 2; *P* = 0.46), which still showed no significant difference between these two groups (Figure 4(b)).

**Figure 5:**
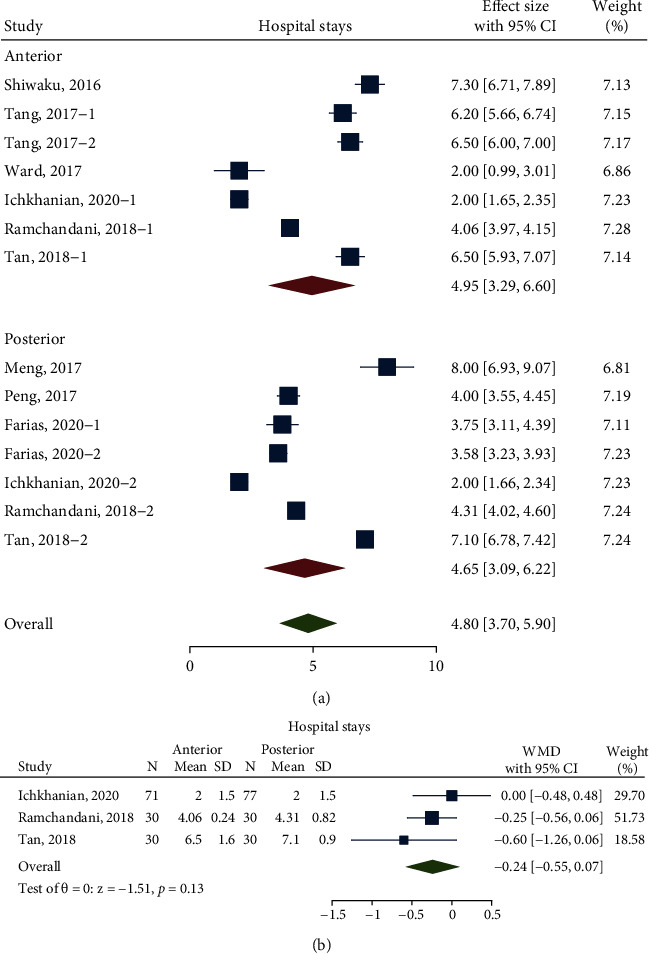
(a) Forest plot, Comparison of hospital stays before balancing baseline; (b) Forest plot, hospital stays difference between anterior and posterior approaches after balancing baseline Labels 1 and 2 were sectionalizations inside the study. They grouped these factors as follows: preoperative intervention/non-preoperative intervention (Tang, 2017), FTM/CM (Duan, 2017), Chagas/idiopathic (Farias, 2020), and anterior/posterior (Ramchandani, 2018; Tan, 2018).

With the meta-analysis of 9 studies including 536 patients (303 patients in the anterior approach and 233 patients in the posterior approach), the hospital stays of the anterior approach was 4.95 days (95% CI 3.29 to 6.60 days; *n* = 6; *I*^2^ 99.23%) and that of the posterior approach was 4.65 days (95% CI 3.09 to 6.22 days; *n* = 6; *I*^2^ 99.07%), which showed no significant difference (*P* = 0.80) (Figure 5(a)). After balancing the characteristics of the studies, the pooled WMD was -0.24 days (95% CI -0.55 to 0.07 days; *I*^2^ 30.85%; *n* = 3; *P* = 0.13), which still showed no significant difference between these two groups (Figure 5(b)).

Compared to the previous study, there were several new studies included in our meta-analysis, and we updated these indexes mentioned above as supplements. In particular, we analyzed the difference between pre- and post-POEM (LES pressure and Eckardt scores), which would be more precise. Additionally, the outcomes as follows were analyzed, and the results are consistent with the previous study: the overall clinical success after POEM with a follow-up time at 12 months and >12 months both showed no obvious difference between the anterior approach and posterior approach, as detailed in Tables 3 and 4 and Supplementary Fig. 1-2. The pooled occurrence of GERD events after POEM and the pooled occurrence of adverse events after POEM did not show a difference between the anterior approach and posterior approach in our analysis. Additionally, it seemed that the procedure time of the posterior approach did not differ from that of the anterior approach in statistics (anterior vs. posterior: 78.33 vs. 70.46 mins; *P* = 0.53). All results are summarized in Tables 3 and 4 and Supplementary Fig. 3-5.

Publication bias was evaluated for the included studies. A funnel plot was used to perform the analysis for our primary outcomes (Supplementary Fig. 7). No publication bias was identified in the results of Eckardt scores and clinical success at 12 months. However, we found publication bias in the results of LES pressure and clinical success > 12 months. Further analysis was conducted by the trim and fill method and confirmed that the trend of pooled effects was similar.

## 5. Discussion

POEM has been more widely used over the past decade in the treatment of achalasia. Several studies have confirmed the safety and efficiency of POEM [10, 11]. A prior study showed that the rate of postprocedure GERD of POEM, clinical success, and adverse events was almost the same in both anterior and posterior approaches [14]. However, these outcomes are quantitative objective indicators. This study analyzed objective indicators to obtain more quantitative and objective outcomes and update the analysis data.

In this study, with a total of 1261 patients from 19 studies, no significant differences between the anterior group and posterior group in terms of the pooled difference in LES pressure before and after POEM, the pooled difference in Eckardt scores before and after POEM, overall clinical success after POEM at 12 months and >12 months, the length of total myotomy, hospital stays, the pooled occurrence of GERD events after POEM, and the pooled occurrence of adverse events were identified.

From this study, the pooled differences in LES pressure of the anterior approach and posterior approach were -24.56 mmHg and -20.14 mmHg, respectively, with a pooled WMD of -1.56 mmHg (*P* = 0.19). In addition, the pooled differences in Eckardt scores of the anterior approach and posterior approach were -5.83 and -6.07, respectively, with a pooled WMD of 0.08 (*P* = 0.66). There was no heterogeneity with the pooled WMD for the pooled difference in LES pressure and Eckardt scores.

As in a previous study [14], the overall clinical success after POEM at the 12-month follow-up and >12-month follow-up, occurrence of GERD events after POEM, and adverse events were similar in anterior myotomy and posterior myotomy. In addition, the length of total myotomy (anterior vs. posterior: 12.30 cm vs. 10.81 cm), hospital stays (anterior vs. posterior: 4.95 vs. 4.65 days), and procedure time (anterior vs. posterior: 78.33 min vs. 70.46 min) in the anterior approach seemed to be comparable to those in the posterior approach. This study further confirms the safety of POEM, and the influence of anterior and posterior approaches on POEM is not significant.

At present, only 2 meta-analyses comparing POEM via the anterior approach and posterior approach have been published [14, 51]. The results reported in this study differed from the results in the latest meta-analysis [14]. Compared with the latest study, two new articles were included in this study [34, 44]. One of the new articles was a follow-up study of the RCT [46]. Additionally, LES pressure and Eckardt scores were added as the primary outcomes, which were quantified indicators. Thus, the results would be more objective. Furthermore, the length of total myotomy and hospital stays were also compared between the anterior and posterior approaches because these two indicators may affect the choice of approach.

For the other meta-analysis [51], the methods and reported outcomes in this meta-analysis are obviously different from those in this study. The earlier meta-analysis only included four RCTs with 488 patients to compare the efficiency of anterior and posterior myotomy. The clinical success, incidence of GERD after POEM, LES pressure, and total operation time did not differ between anterior and posterior myotomy, which was consistent with the findings of this study. However, this meta-analysis indicated that anterior myotomy was associated with a shorter hospital stays, while posterior myotomy had fewer adverse events, lower risk, and shorter incision closure time, which were different from the outcomes in this study. In this study, the length of total myotomy, hospital stays, pooled occurrence of GERD events after POEM, and pooled occurrence of adverse events did not show significant differences between the anterior and posterior approaches. These different results may be attributed to the different quantities and types of included articles. This study included 1261 patients from 19 studies consisting of 3 RCTs and 16 cohorts, which contained a larger population, and the result might be more convincing.

There was no significant difference in procedure time between the anterior and posterior approaches. However, according to theoretical analysis, the endoscope in the posterior approach can fit the working channel better and shorten the incision closure time [14]. Nevertheless, it seems that the length of total myotomy is not affected by the anterior or posterior approach, although the posterior approach provides a better alignment of the endoscopic accessories with the channel of the endoscope. Thus, the hospital stays would not be influenced by the shorter procedure time. However, these outcomes may be influenced by factors such as operator experience, level of health care facility, and patient age, as the heterogeneity is high.

Regarding postprocedure GERD, both this study and a previous study found no difference between the anterior approach and the posterior approach, which is inconsistent with a theoretical analysis: the anterior approach has a lower risk of damage to sling muscle fibers and the angle of His, which might be more beneficial to the antireflux mechanism of the esophagus [5, 14]. This may be due to different skill levels of operators, different lifestyles of patients, and partial or full thickness myotomy. Thus, more studies with head-to-head comparisons between anterior and posterior myotomy are needed.

There are several strengths of this study. A systematic literature search was conducted, with clear inclusion criteria, careful exclusion of redundant studies, inclusion of good-quality studies, detailed extraction of data, and strict evaluation of study quality. This is also the first meta-analysis to compare the difference in LES pressure and Eckardt scores, length of total myotomy, and hospital stays between anterior and posterior approaches.

There are limitations in this study, and some of these are unavoidable. First, most of the studies were observational studies, although 4 of them were performed using a prospective cohort, and 3 RCTs were included. None of the studies were representative of the general population or community practice. These factors have affected the quality of evidence. Second, heterogeneity was identified in several comparisons, including the pooled difference in LES pressure and Eckardt scores. However, there was no heterogeneity with the pooled WMDs in the direct comparison, and it revealed the same outcomes. Thus, the result could be confirmed. The reason for the observed heterogeneity based on the metaregression analysis and sensitivity analysis was not found. Thus, the observed heterogeneity may be related to the difference in the operators' experience and the institutional policy. Since then, studies with large samples and multicenter RCTs have been excluded.

Despite these limitations, this meta-analysis demonstrates that the terms of the pooled difference in LES pressure and Eckardt scores, clinical success, length of total myotomy, hospital stays, GERD, adverse events, and procedure time seemed to be similar for both the anterior and posterior approaches. Further prognosis after POEM via anterior and posterior approaches needs to be studied in the future.

## Figures and Tables

**Table 1:** Characteristics of included studies.

Study	Country	Type of study	Site of myotomy	Age, mean/median (range/SD)	Patients(*n*)	Gender (male/female)	Follow-up (months)	Achalasia(I/II/III)	Course of disease (months)
Hungness et al. [26]	USA	Retrospective (cohort)	Anterior	52.9 (18)	112	68/44	29 (11)	25/58/20	NA
Shiwaku et al. [27]	Japan	Prospective (cohort)	Anterior	48.8 (18.8)	70	41/29	NA	6/55/9	NA
Tang et al. [28]	China	Retrospective (cohort)	Anterior	34.9 (7.7)	22	14/8	12	5/17/0	6.4 (5.4)
				38.5 (11.3)	39	20/19		13/26/0	6.5 (4.8)
Ward et al. [29]	USA	Prospective (cohort)	Anterior	63.0 (17.9)	41	25/16	12	NA	81.6 (117.6)
Werner et al. [30]	Germany, etc.	Retrospective (cohort)	Anterior	44.9 (9–88)	80	43/37	29 (24–41)	24/48/5	NA
Zheng et al. [31]	China	Retrospective (cohort)	Anterior	32.5 (8.36)	26	14/12	12	11/15/0	22.31 (8.31)
de Pascale et al. [32]	Italy	Retrospective (cohort)	Posterior	56 (18–83)	32	20/12	23.7 (12–46.2)	0/31/1	36 (6.0–312)
Duan et al. [33]	China	Retrospective (cohort)	Posterior	43 (14)	70	33/37	30 (24-46)	12/51/7	60.0 (6.0–396.0)
				41 (13)	53	30/23		9/39/5	54.0 (6.0–240.0)
Farias et al. [34]	Brazil	Retrospective (cohort)	Posterior	53.70 (11.74)	20	9/11	12	NA	NA
				44.61 (14.80)	31	15/16	12	NA	NA
Guo et al. [35]	China	Retrospective (cohort)	Posterior	40.7 (15.3)	67	36/31	40.1 (2.8)	13/50/4	94.7 (95.5)
Meng et al. [36]	China	Retrospective (cohort)	Posterior	44.8 (11.6)	32	13/19	25 (11)	5/18/9	24 (12–60)
Peng et al. [37]	China	Retrospective (cohort)	Posterior	37.5 (13.0)	13	8/5	46.2 (4.1)	NA	46.8 (33.6)
Tyberg et al. [38]	USA, etc.	Prospective (cohort)	Posterior	54.2	51	24/27	24.4 (12–52)	13/29/6	134.4
Wang et al. [39]	China	Retrospective (cohort)	Posterior	67.9 (4.3)	21	12/9	21.8	5/16/0	166.8 (140.4)
Zhang and Linghu [40]	China	Retrospective (cohort)	Posterior	43.3 (16–79)	32	16/16	27 (24–51)	0/0/32	24.0 (2.4–336.0)
Ramchandani et al. [41]	India	RCT	Anterior	38 (13.5)	30	15/15	6	5/21/4	22.2 (28.1)
			Posterior	43.9 (15.7)	30	18/12		6/21/3	35.6 (37.6)
Stavropoulos et al. [42]	USA	RCT	Anterior	54.2 (2)	101	52/49	NA	22/58/21	NA
			Posterior	54.8 (1.8)	114	60/54	NA	36/56/22	NA
Tan et al. [43]	China	RCT	Anterior	45.8 (12.2)	31	15/16	15.8 (3.8)	4/26/1	80.4 (80.4)
			Posterior	42.4 (13.3)	32	14/18	15.1 (3.9)	3/28/1	74.4 (86.4)
Ichkhanian et al. [44]	USA, etc.	Prospective (cohort)	Anterior	52.3 (21)	54	29/25	34.5 (6.9)	13/33/8	53.3 (61.4)
			Posterior	51.2 (18)	57	23/34	32.5 (5.2)	4/42/11	50.5 (59.9)

**Table 2:** Metaregression with differences in pre- and postoperative Eckardt scores/LES pressure.

Variate	Meta regression (two-tailed *P* value)	
	Eckardt scores	LES pressure
Length of total myotomy	0.377	0.231
Follow up time	0.678	0.935
Type II proportion in AC	0.058	0.639
Course of disease	0.412	0.297
Prior treatment	0.351	0.279

**Table 3:** Summary of the results before balancing baseline.

Outcome	Effective size (95% CI; *n*; *I*^2^)		
	Anterior	Posterior	*P* value^a^
LES pressures^b^ (mmHg)	-24.56 (-31.29, -17.82; *n* = 5; 96.25%)	-20.14 (-23.44.-16.85; *n* = 8; 94.72%)	0.25
POEM Eckardt^b^	-5.83 (-6.22, -5.45; *n* = 8; 83.15%)	-6.07 (-6.52, -5.62; *n* = 10; 88.93%)	0.44
Clinical success at 12 months^c^ (%)	94 (90, 97; *n* = 8; 46.74%)	95 (92, 98; *n* = 9; 22.00%)	-
Clinical success > 12 months^c^ (%)	86 (78, 94; *n* = 3; 69.24%)	92 (87, 97; *n* = 7; 72.59%)	0.19
Procedure time^d^ (min)	78.33 (56.44, 100.22; *n* = 7; 98.72%)	70.46 (59.05, 81.87; *n* = 10; 98.47%)	0.53
Length of total myotomy^d^ (cm)	12.30 (10.04, 14.56; *n* = 6; 97.70%)	10.81 (9.86, 11.76; *n* = 7; 97.80%)	0.23
Hospital stays^d^ (day)	4.95 (3.29, 6.60; *n* = 6; 99.23%)	4.65 (3.09, 6.22; *n* = 6; 99.07%)	0.80
GERD by EGD^c^ (%)	22 (17, 27; *n* = 9; 58.27%)	16 (12, 21; *n* = 11; 51.38%)	0.11
Adverse events^c^ (%)	2 (0, 7; *n* = 9; 84.88%)	5 (1, 9; *n* = 13; 74.76%)	-

^a^ *P* value of subgroup analysis between anterior and posterior approaches. ^b^Differences in the pre- and postoperative mean of the anterior/posterior approach in the subgroup analysis. ^c^Pooled rate of clinical success at 12 months, clinical success > 12 months, GERD by EGD, and adverse events in subgroup analysis. ^d^Pooled mean procedure time, length of total myotomy, and hospital stays in the subgroup analysis.

**Table 4:** Summary of the results in direct comparison.

Outcome	Effective size (95% CI; *n*; *I*^2^)	*P* value
LES pressures (mmHg)	WMD: -1.56 (-3.90, 0.78; *n* = 3; 0.00%)	0.19
POEM Eckardt	WMD: 0.08 (-0.28, 0.44; *n* = 3; 0.00%)	0.66
Clinical success at 12 months	lnOR: 0.03 (-0.67, 0.74; *n* = 4; 0.00%)	0.92
Procedure time (min)	WMD: 3.41 (-1.14, 7.95; *n* = 4; 0.00%)	0.14
Length of total myotomy (cm)	WMD: 0.36 (-0.60, 1.31; *n* = 2; 67.41%)	0.46
Hospital stays (day)	WMD: -0.24 (-0.55, 0.07; *n* = 3; 30.85%)	0.13
GERD by EGD	lnOR: -0.12 (-0.55, 0.31; *n* = 4; 0.00%)	0.59
Adverse events	lnOR: 0.33 (-0.53, 1.18; *n* = 4; 0.00%)	0.46

## Data Availability

Supplementary Tables 1–2, Supplementary Figs. 1–7, and Appendices 1–2 are available online only (Supplementary Material).

## Supplementary Materials

Supplementary 1 Supplementary Table 1: the NOS quality scores (evaluating the quality of cohort studies).

Supplementary 2 Supplementary Table 2: the Jadad scores (evaluating the quality of RCTs).

Supplementary 3 Supplementary Fig. 1: forest plot and bubble plot and clinical success after POEM at 12 months. (A) Meta-analysis of clinical success at 12 months with the anterior/posterior approach. (B) Metaregression of clinical success after POEM at 12 months with the anterior/posterior approach. (C) Meta-analysis of clinical success after POEM at 12 months in direct comparison with the anterior/posterior approach.

Supplementary 4 Supplementary Fig. 2: forest plot and bubble plot and clinical success after POEM > 12 months. (A) Meta-analysis of clinical success after POEM > 12 months in indirect comparison between anterior and posterior approaches. (B) Metaregression of clinical success after POEM > 12 months and the anterior/posterior approach.

Supplementary 5 Supplementary Fig. 3: forest plot, bubble plot, and procedure time. (A) Meta-analysis of procedure time in indirect comparison between anterior and posterior approaches. (B) Metaregression of the procedure time and anterior/posterior approach. (C) Meta-analysis of procedure time in direct comparison with anterior/posterior approach.

Supplementary 6 Supplementary Fig. 4: forest plot and bubble plot and pooled occurrence of GERD events associated with POEM. (A) Meta-analysis of pooled occurrence of GERD events associated with POEM in indirect comparison between anterior and posterior approaches. (B) Metaregression of pooled occurrence of GERD events and anterior/posterior approach. (C) Meta-analysis of the pooled occurrence of GERD events associated with POEM in direct comparison with the anterior/posterior approach.

Supplementary 7 Supplementary Fig. 5: forest plot and bubble plot and pooled occurrence of adverse events associated with POEM. (A) Meta-analysis of the pooled occurrence of adverse events associated with POEM in the anterior/posterior approach. (B) Metaregression of pooled occurrence of adverse events and anterior/posterior approach. (C) Meta-analysis of the pooled occurrence of adverse events associated with POEM in direct comparison with the anterior/posterior approach.

Supplementary 8 Supplementary Fig. 6: sensitivity analysis for heterogeneous studies in the meta-analysis.

Supplementary 9 Supplementary Fig. 7: funnel plot for publication bias in the meta-analysis (LES pressure, Eckardt scores, and clinical success at 12 months and >12 months).

Supplementary 10 Appendix 1: search strategy.

Supplementary 11 Appendix 2: PRISMA checklist.
