# Supplementary material for: An Updated Meta-analysis: Similar Clinical Efficacy of Anterior and Posterior Approaches in Peroral Endoscopic Myotomy (POEM) for Achalasia
Source: Gastroenterol Res Pract. 2022 Apr 11;2022:8357588. doi: 10.1155/2022/8357588 (PMC9020144; doi:10.1155/2022/8357588)
Supplement: Supplementary 1 — Supplementary Table 1: the NOS quality scores (evaluating the quality of cohort studies). [file 8357588.f1.docx]

Supplementary Table 1. The NOS quality scores (evaluating the quality of cohort studies)

| Study | Selection | | | | Comparability | Outcome | | | Score | Quality |
| --- | --- | --- | --- | --- | --- | --- | --- | --- | --- | --- |
|  | Representativeness of the average adult in community | Cohort size | Information on weight-loss outcomes | Outcome not present at start | Factors comparable between the groups | Adequate clinical assessment | Follow up time of 12-months | Adequacy of follow-up | Max=7 | High>6; Medium 4 to 6; Low<4 |
|  | Population based: 1; multi-center: 0.5;  single-center: 0 | >40 patients: 1;  39 to 20: 0.5; <20: 0 | Information with clarity: 1; information derived from percentage value: 0.5; unclear: 0 | not present: 1; present: 0 | yes: 1;  no: 0 | yes: 1;  no: 0 | yes: 1; not mentioned: 0 | All patients followed up:1;  >50% followed up:0.5; <50% followed up OR not mentioned: 0 |  |  |
| Hungness,  2016 [26] | 0 | 1 | 1 | 1 | 1 | 1 | 1 | 0.5 | 6.5 | High |
| Shiwaku,  2016 [27] | 0 | 1 | 1 | 1 | 1 | 1 | 1 | 0.5 | 6.5 | High |
| Tang,  2017 [28] | 0 | 1 | 1 | 1 | 1 | 1 | 1 | 1 | 7 | High |
| Ward,  2017 [29] | 0 | 1 | 1 | 1 | 1 | 1 | 1 | 1 | 7 | High |
| Werner,  2016 [30] | 0.5 | 1 | 1 | 1 | 1 | 1 | 1 | 0.5 | 7 | High |
| Zheng,  2019 [31] | 0 | 1 | 1 | 1 | 1 | 1 | 1 | 0.5 | 6.5 | High |
| De Pascale,  2017 [32] | 0 | 0.5 | 1 | 1 | 1 | 1 | 1 | 0.5 | 6 | Medium |
| Duan,  2017 [33] | 0 | 1 | 1 | 1 | 1 | 1 | 1 | 0.5 | 6.5 | High |
| Farias,  2020 [34] | 0 | 1 | 1 | 1 | 1 | 1 | 1 | 1 | 7 | High |
| Guo,  2017 [35] | 0 | 1 | 1 | 1 | 1 | 1 | 1 | 0.5 | 6.5 | High |
| Meng,  2017 [36] | 0 | 0.5 | 1 | 1 | 1 | 1 | 1 | 1 | 6.5 | High |
| Peng,  2017 [37] | 0 | 0 | 1 | 1 | 1 | 1 | 1 | 0.5 | 5.5 | Medium |
| Tyberg,  2017 [38] | 0.5 | 1 | 1 | 1 | 1 | 1 | 1 | 0.5 | 7 | High |
| Wang,  2016 [39] | 0 | 0.5 | 1 | 1 | 1 | 1 | 1 | 0.5 | 6 | Medium |
| Zhang,  2017 [40] | 0 | 0.5 | 1 | 1 | 1 | 1 | 1 | 0.5 | 6 | Medium |
| Ichkhanian,  2020 [44] | 0.5 | 1 | 1 | 1 | 1 | 1 | 1 | 0.5 | 7 | High |
