# Supplementary material for: An Updated Meta-analysis: Similar Clinical Efficacy of Anterior and Posterior Approaches in Peroral Endoscopic Myotomy (POEM) for Achalasia
Source: Gastroenterol Res Pract. 2022 Apr 11;2022:8357588. doi: 10.1155/2022/8357588 (PMC9020144; doi:10.1155/2022/8357588)
Supplement: Supplementary 2 — Supplementary Table 2: the Jadad scores (evaluating the quality of RCTs). [file 8357588.f2.docx]

Supplementary Table 2. The Jadad scores (evaluating the quality of RCTs)

| Study | Randomization | Concealment of allocation | Double blinding | Withdrawals and dropouts | Score | Quality |
| --- | --- | --- | --- | --- | --- | --- |
|  | Not randomized or inappropriate method: 0; Described as randomized: 1; The method of randomization was described and it was approprite:2 | Not describe the method of allocation concealment: 0; Described as using allocation concealment method: 1; The method of allocation concealment was described appropriately: 2 | No blind or inappropriate method of blinding: 0; Described as double blind: 1; The method of double blinding was described and it was appropriately: 2 | No describe the follow-up: 0; A description of withdrawals and dropouts: 1 | Max=4 | High 4 to 7; Low 1 to 3 |
| Ramchandani,  2018 [41] | 2 | 0 | 1 | 1 | 4 | High |
| Stavropoulos,  2018 [42] | 2 | 0 | 0 | 1 | 3 | Low |
| Tan,  2018 [43] | 2 | 0 | 0 | 1 | 3 | Low |
