# Supplementary material for: An Updated Meta-analysis: Similar Clinical Efficacy of Anterior and Posterior Approaches in Peroral Endoscopic Myotomy (POEM) for Achalasia
Source: Gastroenterol Res Pract. 2022 Apr 11;2022:8357588. doi: 10.1155/2022/8357588 (PMC9020144; doi:10.1155/2022/8357588)
Supplement: Supplementary 3 — Supplementary Fig. 1: forest plot and bubble plot and clinical success after POEM at 12 months. (A) Meta-analysis of clinical success at 12 months with the anterior/posterior approach. (B) Metaregression of clinical success after POEM at 12 months with the anterior/posterior approach. (C) Meta-analysis of clinical success after POEM at 12 months in direct comparison with the anterior/posterior approach. [file 8357588.f3.docx]

Supplementary Fig. 1. Forest plot and bubble plot, clinical success after POEM at 12-month

A). Meta-analysis of clinical success at 12-month with the anterior/posterior approach


Label1, 2 were sectionalizations inside study. They respectively grouped with such factors: Preoperative intervention/ non-preoperative intervention (Tang, 2017), FTM/ CM (Duan, 2017), Chagas/ Idiopathic (Farias, 2020), Anterior/ Posterior (Ichkhanian, 2020; Ramchandani, 2018; Tan, 2018; Stavropoulos, 2018).

B). Meta-regression of clinical success after POEM at 12-month and with the anterior/posterior approach

“0” : assignment of anterior approach; “1”: assignment of posterior approach

**The overall clinical success rate with 12-month’s follow-up is respectively 94%, 95% in anterior and posterior approach. And Bubble plot showed no relevance between approach and clinical success rate.**

C). Meta-analysis of clinical success after POEM at 12-month in direct comparison with the anterior/posterior approach
