# Supplementary material for: An Updated Meta-analysis: Similar Clinical Efficacy of Anterior and Posterior Approaches in Peroral Endoscopic Myotomy (POEM) for Achalasia
Source: Gastroenterol Res Pract. 2022 Apr 11;2022:8357588. doi: 10.1155/2022/8357588 (PMC9020144; doi:10.1155/2022/8357588)
Supplement: Supplementary 4 — Supplementary Fig. 2: forest plot and bubble plot and clinical success after POEM > 12 months. (A) Meta-analysis of clinical success after POEM > 12 months in indirect comparison between anterior and posterior approaches. (B) Metaregression of clinical success after POEM > 12 months and the anterior/posterior approach. [file 8357588.f4.docx]

Supplementary Fig. 2. Forest plot and bubble plot, clinical success after POEM > 12 months

A). Meta-analysis of clinical success after POEM > 12 months in indirect comparison between anterior and posterior approaches

Label1, 2 were sectionalizations inside study. They respectively grouped with such factors: FTM/ CM (Duan, 2017), Anterior/ Posterior (Ichkhanian, 2020).

B). Meta-regression of clinical success after POEM > 12 months and the anterior/posterior approach

“0”: assignment of anterior approach; “1”: assignment of posterior approach

The overall clinical success rate > 12 months follow-up is respectively 86%, 92% in anterior and posterior approach, there was no statistical difference between the two groups (p=0.19). And Bubble plot intuitively presents the relationship between the two groups. For the limited studies(n=1) report clinical success > 12 months follow-up both in anterior and posterior approach, we have no analysis after balancing baseline for this outcome.
