# Supplementary material for: An Updated Meta-analysis: Similar Clinical Efficacy of Anterior and Posterior Approaches in Peroral Endoscopic Myotomy (POEM) for Achalasia
Source: Gastroenterol Res Pract. 2022 Apr 11;2022:8357588. doi: 10.1155/2022/8357588 (PMC9020144; doi:10.1155/2022/8357588)
Supplement: Supplementary 5 — Supplementary Fig. 3: forest plot, bubble plot, and procedure time. (A) Meta-analysis of procedure time in indirect comparison between anterior and posterior approaches. (B) Metaregression of the procedure time and anterior/posterior approach. (C) Meta-analysis of procedure time in direct comparison with anterior/posterior approach. [file 8357588.f5.docx]

Supplementary Fig. 3. Forest plot, bubble plot, and procedure time

A). Meta-analysis of procedure time in indirect comparison between anterior and posterior approaches

Label1, 2 were sectionalizations inside study. They respectively grouped with such factors: Preoperative intervention/ non-preoperative intervention (Tang, 2017), FTM/ CM (Duan, 2017), Chagas/ Idiopathic (Farias, 2020), Anterior/ Posterior (Ichkhanian, 2020; Ramchandani, 2018; Tan, 2018; Stavropoulos, 2018).

B). Meta-regression of the procedure time and anterior/posterior approach

“0” : assignment of anterior approach; “1”: assignment of posterior approach

**The overall procedure time in anterior and posterior approaches shows no statistical difference (p=0.53). And Bubble plot intuitively presents that there is no obvious relation between approaches and procedure time.**

C). Meta-analysis of procedure time in direct comparison with anterior/posterior approach
