# Supplementary material for: An Updated Meta-analysis: Similar Clinical Efficacy of Anterior and Posterior Approaches in Peroral Endoscopic Myotomy (POEM) for Achalasia
Source: Gastroenterol Res Pract. 2022 Apr 11;2022:8357588. doi: 10.1155/2022/8357588 (PMC9020144; doi:10.1155/2022/8357588)
Supplement: Supplementary 6 — Supplementary Fig. 4: forest plot and bubble plot and pooled occurrence of GERD events associated with POEM. (A) Meta-analysis of pooled occurrence of GERD events associated with POEM in indirect comparison between anterior and posterior approaches. (B) Metaregression of pooled occurrence of GERD events and anterior/posterior approach. (C) Meta-analysis of the pooled occurrence of GERD events associated with POEM in direct comparison with the anterior/posterior approach. [file 8357588.f6.docx]

Supplementary Fig. 4. Forest plot and bubble plot, pooled occurrence of GERD events associated with POEM

A). Meta-analysis of pooled occurrence of GERD events associated with POEM in indirect comparison between anterior and posterior approaches

Label1, 2 were sectionalizations inside study. They all grouped as: Anterior/ Posterior (Ichkhanian, 2020; Ramchandani, 2018; Tan, 2018; Stavropoulos, 2018).

B). Meta-regression of pooled occurrence of GERD events and anterior/posterior approach

“0” : assignment of anterior approach; “1”: assignment of posterior approach

The Pooled occurrence of post-POEM GERD events in anterior and posterior approach shows no statistical difference (p=0.11). And bubble plot intuitively presents that there is no obvious relation between approach and occurrence of post-POEM GERD events.

C). Meta-analysis of the pooled occurrence of GERD events associated with POEM in direct comparison with the anterior/posterior approach
