# Supplementary material for: An Updated Meta-analysis: Similar Clinical Efficacy of Anterior and Posterior Approaches in Peroral Endoscopic Myotomy (POEM) for Achalasia
Source: Gastroenterol Res Pract. 2022 Apr 11;2022:8357588. doi: 10.1155/2022/8357588 (PMC9020144; doi:10.1155/2022/8357588)
Supplement: Supplementary 7 — Supplementary Fig. 5: forest plot and bubble plot and pooled occurrence of adverse events associated with POEM. (A) Meta-analysis of the pooled occurrence of adverse events associated with POEM in the anterior/posterior approach. (B) Metaregression of pooled occurrence of adverse events and anterior/posterior approach. (C) Meta-analysis of the pooled occurrence of adverse events associated with POEM in direct comparison with the anterior/posterior approach. [file 8357588.f7.docx]

Supplementary Fig. 5. Forest plot and bubble plot, pooled occurrence of adverse events associated with POEM

A). Meta-analysis of the pooled occurrence of adverse events associated with POEM in the anterior/posterior approach


Label 1, 2 were sectionalizations inside study. They respectively grouped with such factors: FTM/ CM (Duan, 2017), Chagas/ Idiopathic (Farias, 2020), Anterior/ Posterior (Ichkhanian, 2020; Ramchandani, 2018; Tan, 2018; Stavropoulos, 2018).

B). Meta-regression of pooled occurrence of adverse events and anterior/posterior approach

“0”: assignment of anterior approach; “1”: assignment of posterior approach

**The pooled occurrence of adverse events after POEM is respectively 2%, 4% in anterior and posterior approach. And Bubble plot showed no relevance between approach and occurrence of adverse events after POEM.**

C). Meta-analysis of the pooled occurrence of adverse events associated with POEM in direct comparison with the anterior/posterior approach
