# Supplementary material for: An Updated Meta-analysis: Similar Clinical Efficacy of Anterior and Posterior Approaches in Peroral Endoscopic Myotomy (POEM) for Achalasia
Source: Gastroenterol Res Pract. 2022 Apr 11;2022:8357588. doi: 10.1155/2022/8357588 (PMC9020144; doi:10.1155/2022/8357588)
Supplement: Supplementary 8 — Supplementary Fig. 6: sensitivity analysis for heterogeneous studies in the meta-analysis. [file 8357588.f8.doc]

Supplementary Fig. 6. Sensitivity analysis for heterogeneous studies in the meta-analysis

Fig.6a. LES pressure

Fig.6b. Eckardt scores

Fig.6c. Clinical success at 12-month

Fig.6d. Clinical success >12-months

Fig.6e. Length of total myotomy

Fig.6f. Hospital stay

Fig.6g. Procedure time

Fig.6h. GERD events after POEM

Fig.6i. Adverse events associated with POEM

Label1, 2 were sectionalizations inside study. They respectively grouped with such factors: Preoperative intervention/ non-preoperative intervention (Tang, 2017), FTM/ CM (Duan, 2017), Chagas/ Idiopathic (Farias, 2020), Anterior/ Posterior (Ichkhanian, 2020; Ramchandani, 2018; Tan, 2018; Stavropoulos, 2018).
