# Supplementary material for: An Updated Meta-analysis: Similar Clinical Efficacy of Anterior and Posterior Approaches in Peroral Endoscopic Myotomy (POEM) for Achalasia
Source: Gastroenterol Res Pract. 2022 Apr 11;2022:8357588. doi: 10.1155/2022/8357588 (PMC9020144; doi:10.1155/2022/8357588)
Supplement: Supplementary 9 — Supplementary Fig. 7: funnel plot for publication bias in the meta-analysis (LES pressure, Eckardt scores, and clinical success at 12 months and >12 months). [file 8357588.f9.docx]

Supplementary Fig. 7. Funnel plot for publication bias in the meta-analysis (LES pressure, Eckardt scores, clinical success at 12-month and >12 months)

Fig.7a. LES pressure Fig.7b. LES pressure, Trim and fill method

Fig.7c. Eckardt scores Fig.7d. Clinical success at 12-month

Fig.7e. Clinical success >12 months Fig.7f. Clinical success >12 months
