# Supplementary material for: An Updated Meta-analysis: Similar Clinical Efficacy of Anterior and Posterior Approaches in Peroral Endoscopic Myotomy (POEM) for Achalasia
Source: Gastroenterol Res Pract. 2022 Apr 11;2022:8357588. doi: 10.1155/2022/8357588 (PMC9020144; doi:10.1155/2022/8357588)
Supplement: Supplementary 10 — Appendix 1: search strategy. [file 8357588.f10.docx]

**Appendix 1: Search strategy**

Date range of results 1949-2021

6282 citations located

2324 duplicates removed

3958 items remaining (potential for screening)

**PubMed**

| **Stage** | **Search strategy** |
| --- | --- |
| **#1** | ((((((((((Achalasias, Esophageal[Title/Abstract]) OR (Esophageal Achalasias[Title/Abstract])) OR (Cardiospasm[Title/Abstract])) OR (Cardiospasms[Title/Abstract])) OR (Achalasia[Title/Abstract])) OR (Achalasias[Title/Abstract])) OR (Achalasia, Esophageal[Title/Abstract])) OR (Esophagus achalasia[Title/Abstract])) OR (Megaesophagus[Title/Abstract])) OR (Megaoesophagus[Title/Abstract])) OR (esophagus spasm[Title/Abstract]) |
| **#2** | ((((((((POEM[Title/Abstract]) OR (per oral endoscopic[Title/Abstract])) OR (myotomy[Title/Abstract])) OR (peroral endoscopic myotomy[Title/Abstract])) OR (per-oral endoscopic myotomy[Title/Abstract])) OR (peroral esophageal myotomy[Title/Abstract])) OR (per-oral esophageal myotomy[Title/Abstract])) OR (per-oral myotomy[Title/Abstract])) OR (endoscopic myotomy[Title/Abstract]) |
| **#3** | #1 AND #2 |

| **Stage** | **Search strategy** |
| --- | --- |
| **#1** | 'esophagus achalasia'/exp OR 'megaesophagus'/exp OR 'esophagus spasm'/exp OR achalasia:ti,ab,kw OR achalasias:ti,ab,kw OR 'achalasia, esophageal':ti,ab,kw OR 'esophagus achalasia':ti,ab,kw OR 'esophagus spasm':ti,ab,kw OR megaesophagus:ti,ab,kw OR megaoesophagus:ti,ab,kw OR 'esophageal achalasia':ti,ab,kw |
| **#2** | 'peroral endoscopic myotomy'/exp OR 'peroral esophageal myotomy'/exp OR 'endoscopic myotomy'/exp OR 'per oral endoscopic myotomy':ti,ab,kw OR 'peroral endoscopic myotomy':ti,ab,kw OR 'peroral esophageal myotomy':ti,ab,kw OR 'per-oral esophageal myotomy':ti,ab,kw OR 'per-oral myotomy':ti,ab,kw OR 'endoscopic myotomy':ti,ab,kw |
| **#3** | #1 AND #2 |

**Embase**

| **Stage** | **Search strategy** |
| --- | --- |
| **#1** | (Achalasia): ti,ab,kw or [Esophageal Achalasia] explode all trees |
| **#2** | (POEM): ti,ab,kw OR (per oral endoscopic): ti,ab,kw OR (myotomy): ti,ab,kw OR (peroral endoscopic myotomy): ti,ab,kw OR (per-oral endoscopic myotomy):ti,ab,kw OR (peroral esophageal myotomy): ti,ab,kw OR (per-oral esophageal myotomy): ti,ab,kw OR (per-oral myotomy): ti,ab,kw OR (endoscopic myotomy): ti,ab,kw |
| **#3** | #1 AND #2 |

**Cochrane Library**

| **Stage** | **Search strategy** |
| --- | --- |
| **#1** | TS= (esophageal achalasia OR achalasia OR achalasias OR Achalasia Esophageal OR Achalasias, Esophageal OR Esophageal Achalasias OR Cardiospasm OR Cardiospasms OR Esophagus achalasia OR esophagus spasm OR Megaesophagus OR Megaoesophagus) |
| **#2** | TS= (POEM OR peroral endoscopic myotomy OR per-oral endoscopic myotomy OR peroral esophageal myotomy OR per-oral esophageal myotomy OR per-oral myotomy OR endoscopic myotomy) |
| **#3** | #1 AND #2 |

**Web of science**
